# Veterinary systems biology for bridging the phenotype–genotype gap via computational modeling for disease epidemiology and animal welfare

**DOI:** 10.1093/bib/bbae025

**Published:** 2024-02-10

**Authors:** Rajesh Kumar Pathak, Jun-Mo Kim

**Affiliations:** Department of Animal Science and Technology, Chung-Ang University, Anseong-si, Gyeonggi-do 17546, Republic of Korea; Department of Animal Science and Technology, Chung-Ang University, Anseong-si, Gyeonggi-do 17546, Republic of Korea

**Keywords:** veterinary systems biology, multi-omics, computational models, network analysis, systems vaccinology, drug discovery

## Abstract

Veterinary systems biology is an innovative approach that integrates biological data at the molecular and cellular levels, allowing for a more extensive understanding of the interactions and functions of complex biological systems in livestock and veterinary science. It has tremendous potential to integrate multi-omics data with the support of vetinformatics resources for bridging the phenotype–genotype gap via computational modeling. To understand the dynamic behaviors of complex systems, computational models are frequently used. It facilitates a comprehensive understanding of how a host system defends itself against a pathogen attack or operates when the pathogen compromises the host’s immune system. In this context, various approaches, such as systems immunology, network pharmacology, vaccinology and immunoinformatics, can be employed to effectively investigate vaccines and drugs. By utilizing this approach, we can ensure the health of livestock. This is beneficial not only for animal welfare but also for human health and environmental well-being. Therefore, the current review offers a detailed summary of systems biology advancements utilized in veterinary sciences, demonstrating the potential of the holistic approach in disease epidemiology, animal welfare and productivity.

## INTRODUCTION

The welfare of animals is intertwined with human and environmental welfare. The majority of human diseases originate from animals; therefore, human health is closely linked with animal health and the environment (https://www.fao.org/one-health/en; accessed on 8 February 2023).The bond between livestock and humans has played an important role since the ancient times. The livestock on which the society depends for dairy and other food products are often overlooked. However, they form a bond with their owners (https://cvm.msu.edu/news/perspectives-magazine/perspectives-fall-2018/the-bond-between-humans-and-livestock; accessed on 08/02/2023). Because of their key roles in human nutrition and health as well as the environment, the health and welfare of livestock are of great importance [1]. Therefore, a holistic approach to veterinary research is needed to meet the demands of the growing population in a sustainable manner [2]. Systems biology is a well-established discipline in biomedical science and has a substantial track record of success in solving complex biological problems [3–5]. Given that over 60% of human diseases originate from animals, it is crucial to adopt novel and innovative computational modeling approaches in veterinary science to comprehend the intricate nature of host–pathogen interactions for the sake of animal and human health [6]. To understand the connection between animal, human and environmental health, the concept of One Health has been introduced. It aims to visualize the health of ecosystems comprehensively and in a timely manner, with the goal of achieving optimal health and sustainability for all simultaneously [7, 8]. Consequently, the veterinary science community has embraced these approaches in their research, leading to the emergence of the concept of veterinary systems biology, which primarily focuses on livestock to provide high-quality veterinary services [2].

Systems genetics can help to gain insights into livestock health, productivity and disease epidemiology [2, 9]. Systems genetics is a field that combines genetics, genomics, systems biology and phenomics [9]. The alternation in genotype or phenotype due to biotic factors, such as diseases, or abiotic stresses, like temperature and cold, can increase the mortality rate in livestock [10, 11]. This can be overcome through computational modeling utilizing genotypic and phenotypic data, which can assist in the management and development of protocols for proper nutrition [10, 12]. Besides, we can predict survival by combining genotypic and phenotypic information [13].

It is now possible to conduct a comprehensive analysis through integration of multi-omics datasets generated by genomics, transcriptomics, proteomics, metabolomics and other omics studies with systems biology [2, 14]. However, there are also some disadvantages. It can be technically complex, requiring specialized expertise and computational resources [2]. Additionally, the large-scale data generated by omics technologies can be challenging to analyze, integrate and interpret accurately. Furthermore, the cost of multi-omics analysis can be a limiting factor in livestock research [2, 14–16].

**Figure 1:**
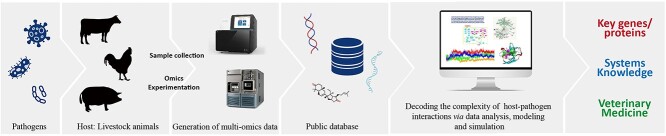
A systems biology approach provides a comprehensive understanding of host–pathogen interactions through the integration of multi-omics data, identifying key genes/proteins and related information to improve the health and welfare of livestock.

The benefits of multi-omics analysis in deciphering certain diseases in livestock are significant. By integrating data from different omics layers, we can gain a more comprehensive understanding of the molecular mechanisms underlying diseases [2]. This integrated approach allows us to identify complex networks, decoding the causes of diseases and visualizing potential molecular targets for drug discovery and developing more effective vaccines [2, 15, 17–19]. Combined multi-omics analysis also improves the accuracy and robustness of biomarker discovery efforts in livestock, leading to more reliable diagnostic tools and predictive models. Ultimately, the use of multi-omics in livestock research can contribute to improved disease management, breeding strategies and overall livestock health and welfare [15, 16, 20–22]. Therefore, the goal of veterinary systems biology analyses is to identify key components associated with the phenotypes of interest using relevant statistical methods and extensive network analysis [23]. It will be necessary to integrate statistical techniques with computational analyses for handling the big data in veterinary science (Figure 1) [14, 23, 24].

Several diseases cause high mortality rates in livestock, resulting in enormous losses to farmers; thus, therapies are urgently needed to suppress disease progression and aid organisms in recovering from abnormal conditions [2, 25].The cost of developing new drugs is high since a drug overdose can cause toxicity and side effects, resulting in long-term safety procedures in clinical studies [25, 26].The study of disease mechanisms and drug responses is facilitated by the analyses of multi-omics data. While single-omics data analysis focuses on a specific aspect and provides a smaller amount of information with lower complexity [14, 25], systemic gene regulation suggests that genes act as a part of complex networks, not alone, to perform cellular functions [25, 27]. A comprehensive analysis and prediction based on complex cellular networks requires the integration of multi-omics datasets, for example, those on transcription factors, genes and their expression products [28, 29]. Therefore, data from omics-based experiments can be used to predict potential molecular interactions in livestock systems and help visualize their dynamic behavior under various conditions and at distinct times with respect to external factors [2, 14]. Such modeling approaches are crucial for building a framework to fill the phenotype–genotype gap in disease epidemiology and for animal welfare using systems biology tools and databases (Table 1).

**Table 1:** Comprehensive list of commonly utilized vet/bioinformatics tools and database resources within the domain of veterinary systems biology including brief descriptions and links

S.N.	Tool/database	Application	Link(s)	Reference(s)
1	R/Bioconductor	Statistical computing and graphics platform and collection of open-source software packages widely used in systems biology for analysis and visualization of multi-omics data.	https://www.r-project.org/ https://www.bioconductor.org/	[30–32]
2	CellDesigner	Graphical modeling and simulation tool for creating and analyzing biological networks.	https://www.celldesigner.org/	[33, 34]
3	Cytoscape	Visualization and analysis of molecular interaction networks, such as protein–protein interactions and metabolic networks.	https://cytoscape.org/	[35]
4	NetworkAnalyzer	Cytoscape application designed to analyze biological networks and compute network topology.	https://apps.cytoscape.org/apps/networkanalyzer	[36]
5	cytoHubba	Cytoscape application employed for investigation of hub nodes and sub-networks within complex interactomes.	https://apps.cytoscape.org/apps/cytohubba	[37]
6	Omics Visualizer	Cytoscape application utilized for the visualization and analysis of several data associated with the same node.	https://apps.cytoscape.org/apps/omicsvisualizer	[38]
7	MATLAB	Mathematical modeling and simulation analysis of biological systems.	https://www.mathworks.com/products/matlab.html	[39]
8	VaxiJen	Tool for prediction of protective antigenicity.	https://www.ddg-pharmfac.net/vaxijen/VaxiJen/VaxiJen.html	[40]
9	NetMHCpan	Tool for prediction of CTL epitope.	https://services.healthtech.dtu.dk/services/NetMHCpan-4.1/	[41]
10	NetMHCIIpan	Tool for prediction of HTL epitope.	https://services.healthtech.dtu.dk/services/NetMHCIIpan-2.1/	[42]
11	ABCpred	Tool for prediction of B-cell epitope.	https://webs.iiitd.edu.in/raghava/abcpred/ABC_submission.html	[43]
12	AllerTOP	Tool for allergenicity prediction.	https://www.ddg-pharmfac.net/AllerTOP/	[44]
13	AllergenFP	Tool for allergenicity prediction.	https://ddg-pharmfac.net/AllergenFP/	[45]
14	JCat	Codon adaptation tool.	https://www.jcat.de/Start.jsp	[46]
15	AlphaFold	Protein three-dimensional structure prediction tool.	https://github.com/google-deepmind/alphafold	[47]
16	AutoDock Vina	Tool used for molecular docking and virtual screening.	https://vina.scripps.edu/	[48, 49]
17	pkCSM	ADMET prediction tool.	https://biosig.lab.uq.edu.au/pkcsm/	[50]
18	Gromacs	Tool used for MD simulation.	https://www.gromacs.org/	[51, 52]
19	SnapGene	Tool used for in silico cloning and other application.	https://www.snapgene.com/	[53, 54]
20	C-ImmSim	Tool used for immune response simulation using vaccine construct.	https://kraken.iac.rm.cnr.it/C-IMMSIM/	[55]
21	BioGRID	Curated database on protein–protein, genetic and chemical interactions in various organisms, including livestock animals, can be utilized for network analysis and identification of drug targets.	https://thebiogrid.org/	[56]
22	KEGG	Holds information on pathways, networks and functions of genes and proteins in various organisms for systems biology analysis.	https://www.genome.jp/kegg/	[57]
23	STRING	Provides data on protein–protein interactions, functional associations and regulatory networks for various applications.	https://string-db.org/	[58]
24	Reactome	Curated pathway database offers user-friendly bioinformatics tools that enable the visualization, interpretation and analysis of pathways, supporting several research activities including genome analysis, modeling and systems biology.	https://reactome.org/	[59]
25	BioModels	Holds mathematical models of biological systems, which can be utilized for modeling and simulation analysis, testing hypotheses and designing new experiments.	https://www.ebi.ac.uk/biomodels/	[60]
26	Sequence Read Archive	Stores high-throughput sequencing data and provides a platform for researchers to submit their own data and access data submitted by others for comprehensive analysis.	https://www.ncbi.nlm.nih.gov/sra	[61]
27	Gene Expression Omnibus (GEO)	GEO is a database hosted by NCBI that holds gene expression data.	https://www.ncbi.nlm.nih.gov/geo/	[62]
28	ArrayExpress	A database holds high-throughput functional genomics data.	https://www.ebi.ac.uk/biostudies/arrayexpress	[63]
29	Protein ANalysis THrough Evolutionary Relationships (PANTHER)	PANTHER is a database supporting high-throughput analysis.	https://www.pantherdb.org/	[64]
30	MetaCyc	A database contains experimentally elucidated metabolic pathways.	https://metacyc.org/	[65, 66]
31	Molecular INTeraction database (MINT)	MINT contains information on protein–protein interactions determined by experimental methods from scientific literature.	https://mint.bio.uniroma2.it/	[67]
32	BRENDA	A database contains functional data related to enzymes.	https://www.brenda-enzymes.org/	[68]
33	Protein Data Bank (PDB)	PDB holds experimentally determined macromolecular structures alongside predicted structure models.	https://www.rcsb.org/	[69]
34	AlphaFold Protein Structure Database	A database holds modeled 3D protein structures.	https://alphafold.ebi.ac.uk/	[70]
35	ZINC	A database of commercially accessible small molecules/compounds for molecular docking and structure-based virtual screening.	https://zinc20.docking.org/ https://cartblanche22.docking.org/	[71, 72]

### Integrative systems biology (top-down approach) and predictive systems biology (bottom-up approach)

Integrative and predictive systems biology are powerful approaches and have tremendous potential for the investigation of novel information and visualization of biological systems [73–76]. Several articles have discussed the fundamental concepts about these approaches [4, 14]. Basically, we analyze and integrate the data generated by genomics, transcriptomics, proteomics and other omics-based studies to make novel discoveries under integrative systems biology. Predictive systems biology, on the other hand, aims to construct a computational model with known components identified through integrative systems biology to predict the dynamic nature of biological systems to develop future strategies for the animal welfare [2, 14].

## SYSTEMS BIOLOGY FOR THE INTEGRATION OF MULTI-OMICS DATA TO BRIDGE THE PHENOTYPE–GENOTYPE GAP

There has been an explosion of high-throughput techniques in recent years. High-throughput experiments are particularly useful in obtaining a comprehensive picture of the physiological traits such as skin temperature of livestock in response to dynamic environmental changes [23]. These innovations and next-generation technology have transformed veterinary science in the era of big data. However, a general framework is still lacking for linking physiological traits to deoxyribonucleic acid variants [23, 77]. In order to better understand how genotype is translated into phenotype, systems biology approaches can be utilized. An effective method for linking a particular genetic background to a disease or a trait is to conduct genome-wide association studies (GWAS). Nevertheless, single-omics data offer limited insights into biological mechanisms, and to enhance the precision of predicting the link between genotype and phenotype, it is essential to incorporate multi-omics data [23, 78, 79]. Several studies have demonstrated the utility of systems biology in integrating multi-omics data. Our lab has previously conducted a study that utilized GWAS and network analysis to identify specific chromosomal regions and potential candidate genes that could have an impact on milk production phenotypes in a population of Korean Holstein cattle [80]. Naserkheil *et al.* (2022) identified the key genes and pathways via multi-omics analysis for the prevention and treatment of mastitis in dairy cattle [79]. In addition, a recent report provides a comprehensive resource for studying functional genomics in cattle and highlights the importance of integrating multi-omics data for a more complete understanding of livestock systems [81].

### Multi-omics data integration methods

The integration of multi-omics data involves gathering high-quality data related to genomics, transcriptomics, proteomics, metabolomics and other omics [2, 21, 82]. This is followed by data pre-processing and quality control analysis to remove errors and ensure compatibility besides annotation and identification of genes, proteins, metabolites and other components along with predicting connections among them [21, 83]. Additionally, several computational modeling approaches can be applied to investigate the functional correlations [82, 83]. Network and pathway analysis approaches aid in the construction of networks for visualizing interactions among genes, proteins and metabolites and in identifying hub nodes and pathways that can assist in the development of disease management strategies [79, 84, 85]. Moreover, machine learning approaches can be utilized to integrate multi-omics datasets. These integrated datasets can be used to train models that help predict disease outcomes and serve as support systems for the development of disease prevention strategies [86–88]. Recently published multi-omics integration and network analysis studies reveal *CXCR1, HCK, IL1RN, MMP9, S100A9, GRO1* and *SOCS3* as hub genes. These genes could be promising candidates for understanding mastitis susceptibility and resistance in dairy cattle [79]. Multi-omics integration, coupled with artificial intelligence, can also guide drug discovery research [89, 90].

### Network construction and analysis

Systems biology can aid in constructing and analyzing the networks of molecular interactions in livestock for the identification of potential drug targets and key genes [91, 92]. By integrating diverse types of omics data, such as genomic, transcriptomic and metabolomic data, these networks can capture the complexity of biological systems and reveal key regulatory factors [2, 21]. Network analysis methods can then be used to identify key molecular players (hub genes) and guide the development of evidence-based management strategies for improving animal health and welfare [20, 24]. Recent studies based on network analysis identified the hub genes *DCXR, MMP15* and *MMP17* associated with subacute ruminal acidosis disorder in dairy cows [93]. Besides, several studies demonstrated the power of network analysis in livestock research for an increased understanding of bovine respiratory disease, PRRS in pigs and hoof disease [14, 20, 24, 93]. Therefore, it has the potential to improve our understanding of veterinary systems biology, which will facilitate the efficient management and prevention of diseases [2].

### Modeling and simulation analysis

Modeling and simulation analysis using systems biology can provide a comprehensive understanding of biological systems [33, 94, 95]. In the context of livestock health, these models can aid in identifying key components involved in disease progression. These models can also help identify potential therapeutic targets and guide the development of veterinary medicine for improving livestock health and welfare [96, 97]. It is important to ensure that the model behaves like biological systems [98]. Several steps are involved in the model validation process, including literature mining, comparing the model output with experimental data, sensitivity analysis, optimizing the model through parameter estimation, conducting robustness testing and refining the model if errors are observed by adjusting parameters, reaction kinetics, to ensure accurate predictions [99–101]. A previous study established a network-based model for simulating the transmission of porcine reproductive and respiratory syndrome virus (PRRSV) between farms. The study demonstrated the potential of the model for identifying high-risk farms and evaluating the effectiveness of different control measures for PRRSV transmission [102]. Another study utilizing pharmacokinetic and pharmacodynamic modeling and simulation of the antibiotic cyadox against *Clostridium perfringens* in swine suggested that cyadox had a strong antibacterial effect and might be a promising alternative for the treatment of *C. perfringens* infections after further validation in clinical studies [103].

## SYSTEMS BIOLOGY APPLIED TO DISEASE EPIDEMIOLOGY AND ANIMAL WELFARE

Systems biology can provide valuable insights into disease epidemiology in livestock [5, 104]. By integrating diverse types of omics data, systems biology can identify key genes and pathways involved in disease susceptibility, transmission and spread. This can aid in predicting effective interventions and guide the management practices for improving animal health and welfare [14, 104]. Furthermore, systems biology can provide a comprehensive view of the interplay between environmental factors, animal physiology and behavior and of how these factors can impact animal welfare [105]. This can guide the development of evidence-based management strategies for promoting animal welfare in livestock production systems [2]. An integrative systems biology study identified several key genes, such as *PRDX5*, *RAB5C*, *ACTN4*, *SLC25A16*, *MAPK6*, *CD53*, *NCKAP1L*, *ARHGEF2*, *COL9A1* and *PTPRC*, as well as pathways involved in the development and progression of mastitis, providing potential targets for future research and therapeutics [18]. Another study identified new treatment options by repurposing existing drugs Glibenclamide, Ipratropium, Salbutamol and Carbidopa for veterinary medicine against *Escherichia coli* mastitis [106]. A system-based analysis at 6, 12 and 24 h post-infection with African swine fever virus (ASFV) revealed 1677, 2122 and 2945 upregulated differentially expressed genes (DEGs), as well as 933, 1148 and 1422 downregulated DEGs, respectively, compared to mock-infected groups. The findings indicated a significant impact of ASFV infection on host metabolism pathways, immune responses and cell death pathways [107]. Previous study conducted in our lab on PRRSV infection in pigs utilized integrated time-serial transcriptome networks to reveal common innate immune responses across different tissues and identify tissue-specific adaptive immune responses. The systems biology approach uncovered distinct expression patterns, such as antiviral signaling at 3 days post-infection, influenza A-like responses in the lungs and downregulated AMP-activated protein kinase (AMPK) signaling in bronchial lymph nodes. These findings offer comprehensive insights into understanding PRRSV infection and help in developing strategies for vaccine development [24]. Therefore, systems-based approaches coupled with vetinformatics provide key insights to understand pathogenesis and highlight potential targets for developing therapeutics for animal health and welfare [2, 108].

### Systems immunology, network pharmacology, drug discovery and repurposing

Systems immunology and network pharmacology are powerful approaches for drug discovery and repurposing in livestock [2, 93]. By integrating diverse types of omics data, these approaches can identify key components and pathways involved in immune system function and drug response [22, 109]. This can aid in identifying novel drug targets and repurposing existing drugs for use in livestock [110, 111]. Furthermore, network pharmacology can predict drug interactions and side effects, enabling the selection of safe and effective drug combinations for complex diseases [93, 112]. These approaches have the potential to accelerate the development of new therapies for improving livestock health and production, while reducing the use of antibiotics. Recent studies identified several compounds with promising antiviral properties, highlighting the potential of natural products in the development of antiviral therapeutics against PRRSV [17, 113].

### Systems vaccinology and immunoinformatics

**Figure 2:**
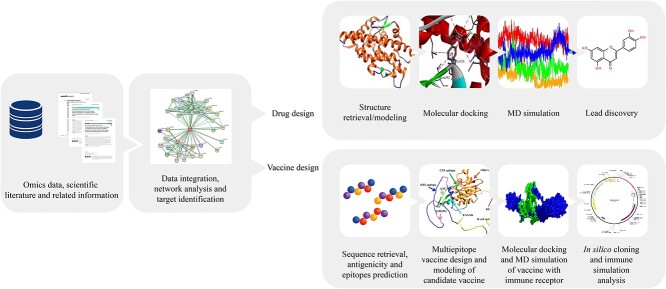
Implementation of systems biology for the identification of potential drug and vaccine candidates targeting livestock diseases.

Systems vaccinology and immunoinformatics are emerging fields that have the potential to revolutionize vaccine development for livestock [19, 114, 115]. By integrating diverse types of omics data and computational modeling, these approaches can identify key genes and pathways involved in immune system response to vaccines [116–118]. This can aid in identifying optimal vaccine formulations, delivery strategies and adjuvants for inducing protective immunity in livestock [119–121]. Furthermore, immunoinformatics can predict vaccine efficacy, safety and potential side effects, enabling the selection of safe and effective vaccine candidates [19]. Recent studies have demonstrated the potential of immunoinformatics-based approaches for the design of multiepitope vaccines against infectious bursal disease virus for chicken [122] and mastitis for cattle [19]. Researchers utilized immunoinformatics-based analysis to explore the impact of the bovine viral diarrhea virus (BVDV). They identified key virulent proteins in BVDV1 and BVDV2, highlighting the differences in antigenicity. The study suggests sub-genotypes (1a, 1f, 1k, 2a and 2b) as potential candidates for future vaccine development [123]. A recent study investigates the worldwide economic implications of the lumpy skin disease virus (LSDV) in cattle. The researchers developed a multi-epitope vaccine by analyzing the LSDV proteome, identifying four antigenic, non-homologous and highly conserved proteins. The analysis reveals the promising potential of the modeled subunit vaccine candidate, demonstrating its interaction with the TLR4 receptor and marking significant progress in the development of an LSDV subunit vaccine [124]. Besides, several studies established the use of these approaches and pipelines in designing vaccine candidates against diseases in humans as well as in other animals [108, 125]. Therefore, these approaches have the potential to improve vaccine design and accelerate the development of new vaccines for preventing infectious diseases in livestock (Figure 2).

## DISCUSSION

Veterinary systems biology coupled with vetinformatics improve our understanding of the complex interactions within livestock biological systems and provide useful information for improving animal health and welfare [2, 5]. Essentially, vetinformatics addresses challenges within the field of veterinary science by employing computational methods supported by bioinformatics resources [2]. While bioinformatics is a broad discipline encompassing various areas of science and technology, vetinformatics specifically focuses on veterinary science [2, 108]. It is a subject with applications in specific research areas, similar to cropinformatics, chemoinformatics, biomedical informatics, etc. [108]*.* Veterinary systems biology takes a holistic approach to decoding livestock systems, emphasizing the comprehensive understanding of complete biological systems rather than studying individual genes or proteins, supported by vetinformatics resources [2, 14, 126]. Integrating genetics, genomics and phenomics with systems models, we can gain a deeper understanding of the complex relationship between genes, their functions and the resulting phenotypes or observable traits [9, 127, 128]. This interdisciplinary approach allows for a comprehensive analysis of genetic variations and their impact on the overall functioning and characteristics of organisms using systems genetics and biology [2, 9]. Therefore, the key advantage of veterinary systems biology is its ability to integrate multi-omics data, including genomics, transcriptomics and metabolomics data, and other omics datasets, to construct comprehensive models to study disease epidemiology and dissect the intricate molecular mechanisms of host–pathogen interactions crucial to animal welfare [14, 23, 76]. These models can be used to simulate and visualize various biological scenarios and make predictions using systems biology tools and databases, aiding in the identification of key genes/proteins and pathways associated with disease resistance and susceptibility, as well as their interplay with external stimuli, for understanding the pathophysiology and how these factors can impact animal welfare [25, 60, 76].

Furthermore, we emphasize the tremendous potential of veterinary systems biology for the discovery and design of novel therapeutics for improving livestock health [17, 113, 129]. Systems biology includes modeling and simulation of biological systems to identify potential therapeutic targets [24]. Therapeutic targets can be utilized for high-throughput screening of small molecules. This can be achieved through various techniques, including target structure modeling, molecular docking, virtual screening, molecular dynamics (MD) simulations and binding energy calculations. These methods help in investigating lead compounds for drug development [103]. Furthermore, these therapeutic targets can also play a crucial role in developing next-generation vaccines. This involves predicting antigenicity, epitopes, designing vaccine candidates and conducting immune simulation analyses to ensure long-term protection. Such approaches contribute to improving animal welfare in livestock production systems [19].

Ultimately, the aim of this review article was to emphasize how veterinary systems biology revolutionizes our understanding of livestock biology for bridging the gap between phenotype and genotype and to guide the development of effective management strategies to improve the health and well-being of livestock [2]. As such, these approaches have the potential to contribute to more sustainable and efficient livestock production systems, while reducing the use of antibiotics, and maintaining human and environmental health as well [14, 15, 19, 25, 105, 130].

## CONCLUSION

Bridging the gap between the computational world and veterinary practice in the field holds tremendous potential for advancing veterinary systems biology. Veterinary systems biology, coupled with vetinformatics resources, and their utilization in veterinary biochemistry, anatomy, physiology, pharmacology and toxicology, microbiology, pathology, parasitology, genetics and breeding and epidemiology, as well as animal nutrition and poultry science, can revolutionize the diagnostics, treatment and overall veterinary practice. However, modeling and simulation of biological systems face several challenges due to biological complexity, integration of diverse data sets and different scales and formats. Developing accurate models and their validation that can predict the behavior of systems remain challenging. Conquering these challenges requires interdisciplinary collaboration to develop and improve computational methods as well as refinement in experimental techniques. This will enhance the accuracy of computational predictions and aid in veterinary research as well as drug discovery. Therefore, the implementation of these advancements will lead to improved animal welfare, optimized health-care outcomes and a brighter future for livestock productivity and sustainability.

**Key Points**

- The bond between livestock and humans has played an important role since ancient times.
- More than 60% of human diseases originate from animals, posing threats to human life.
- Veterinary systems biology provides a comprehensive understanding of host–pathogen interactions through the integration of multi-omics data.
- Veterinary systems biology aids in the identification of drug and vaccine candidates targeting livestock diseases.
- The well-being of livestock is interconnected with human and environmental health.

## Author Biographies

**Rajesh Kumar Pathak** is a postdoctoral researcher at the Department of Animal Science and Technology, Chung-Ang University, Anseong-si, Republic of Korea. His research topic includes bioinformatics and systems biology.

**Jun-Mo Kim** is an associate professor of functional genomics and bioinformatics at the Department of Animal Science and Technology, Chung-Ang University, Anseong-si, Republic of Korea. His research focuses on applying bioinformatics and systems biology approaches to decipher complex mechanism in livestock diseases.
